# Insight into Molecular Interactions of Two Methyl Benzoate Derivatives with Bovine Serum Albumin

**DOI:** 10.3390/ijms222111705

**Published:** 2021-10-28

**Authors:** Karolina Baranowska, Michał Mońka, Piotr Bojarski, Marek Józefowicz

**Affiliations:** Institute of Experimental Physics, Faculty of Mathematics, Physics and Informatics, University of Gdańsk, Wita Stwosza 57, 80-308 Gdańsk, Poland; karolina.baranowska@phdstud.ug.edu.pl (K.B.); michal.monka@ug.edu.pl (M.M.); piotr.bojarski@ug.edu.pl (P.B.)

**Keywords:** inclusion complex, bovine serum albumin, tryptophan, excited-state intramolecular proton transfer, supramolecular interactions

## Abstract

The nature and mechanisms of interaction between two selected methyl benzoate derivatives (methyl *o*-methoxy *p*-methylaminobenzoate–**I** and methyl *o*-hydroxy *p*-methylaminobenzoate–**II**) and model transport protein bovine serum albumin (BSA) was studied using steady-state and time-resolved spectroscopic techniques. In order to understand the role of Trp residue of BSA in the **I**-BSA and **II**-BSA interaction, the effect of free Trp amino acid on the both emission modes (LE–locally excited (**I** and **II**) and ESIPT–excited state intramolecular proton transfer (**II**)) was investigated as well. Experimental results show that the investigated interactions (with both BSA and Trp) are mostly conditioned by the ground and excited state complex formation processes. Both molecules form stable complexes with BSA and Trp (with 1:1 stoichiometry) in the ground and excited states. The binding constants were in the order of 10^4^ M^−1^. The absorption- and fluorescence-titration experiments along with the time-resolved fluorescence measurements show that the binding of the **I** and **II** causes fluorescence quenching of BSA through the static mechanism, revealing a 1:1 interaction. The magnitude and the sign of the thermodynamic parameters, Δ*H*, Δ*S*, and Δ*G*, determined from van’t Hoff relationship, confirm the predominance of the hydrogen-bonding interactions for the binding phenomenon. To improve and complete knowledge of methyl benzoate derivative-protein interactions in relation to supramolecular solvation dynamics, the time-dependent fluorescence Stokes’ shifts, represented by the normalized spectral response function *c(t)*, was studied. Our studies reveal that the solvation dynamics that occurs in subpicosecond time scale in neat solvents of different polarities is slowed down significantly when the organic molecule is transferred to BSA cavity.

## 1. Introduction

During the past decade, considerable attention has been focused on characterization of noncovalent interactions between a macrocyclic host (chemical and/or biological) and different guest molecules (especially drugs and/or small bioactive molecules) [1,2,3]. Cyclodextrins (α, β and γ), calixarenes, cucurbiturils, pillararenes, crown ethers and cyclophanes are one of such chemical organized assemblies possessing a hydrophobic nanocavity, which can accommodate guest molecules [4,5,6]. The understanding of the host–guest interactions is highly desirable for at least two main reasons. First, the explanation of the photochemical and photophysical processes leading to the formation of host–guest inclusion complex is interesting from a spectroscopic point of view, because the incorporation of the molecule into the chemical or biological nanocavity creates a new chemical system with significantly different spectroscopic properties [7,8]. Conversely, owing to complexation ability, chemical and biological cavities have received widespread attention for applications in medicine, agriculture, nanotechnology, cosmetics, food and pharmaceutical industries [9,10,11,12,13,14]. Among various representatives of chemical and biological macromolecules, nontoxic cyclodextrins (chemical) and human as well as bovine serum albumin (biological) are the most investigated ones because of their use in medicine as drug delivery carriers (cyclodextrins) and transport proteins (serum albumins) [15,16,17,18,19]. 

**Scheme 1:**
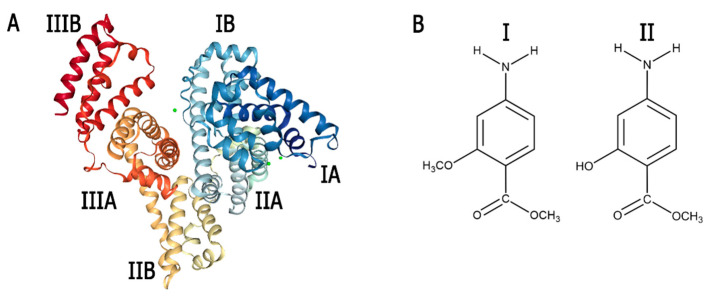
Structure of bovine serum albumin (BSA) (**A**) and methyl benzoate derivatives (methyl *o*-methoxy *p*-methylaminobenzoate–**I**, methyl *o*-hydroxy *p*-methylaminobenzoate–**II**) (**B**).

Serum albumins, the most abundant proteins in the blood plasma, play a significant role in the transport of both exogenous and endogenous compounds. Several experimental and theoretical studies have shown that human serum albumin (HSA) and bovine serum albumin (BSA) present an ideal model of serum carrier protein, and therefore they are most widely used as model proteins in evaluating small organic molecules (drug)−protein interactions [15,20,21,22,23]. BSA finds a wider range of applications because of its (i) low cost, (ii) high water solubility (iii) almost 80% sequence homology with HSA and (iv) widespread availability in a pure form. The crystal structure of BSA shows three domains (I, II, III), which are divided into two subdomains, named A and B [24,25] (Scheme 1A). The drug-binding sites are located in hydrophobic cavities in subdomains IIA and IIIA. Although HSA and BSA exhibit similar conformation properties, their spectroscopic properties are different. This is because BSA has two tryptophan residues that possess intrinsic fluorescence (Trp 134 and Trp 212), whereas HSA has only one residue (Trp 214) [26,27]. Trp 212 is located within a hydrophobic binding pocket of the protein and Trp 134 is located on the surface of the molecule [24].

We have recently undertaken spectroscopic investigations of the some methyl benzoate derivatives which, on the one hand, are interesting from the spectroscopic point of view (they are capable of exhibiting Twisted Intramolecular Charge Transfer- and/or Excited State Intramolecular Proton Transfer-type behaviour) and, conversely, they have potential in applications e.g., TICT and ESIPT fluorescence probes, metal ion sensors, active materials in dry xerographic toners or lasing medium in proton transfer lasers [28,29,30,31,32]. Our scientific attention has been focused not only on the description of the TICT and ESIPT processes of organic molecules in different organic solvents and heterogeneous systems, but also in aqueous solution in the presence of the supramolecular compounds (α-, β- and γ-cyclodextrins, cucurbit[7]uril) [33,34,35,36,37]. We have shown that photochemical and photophysical properties of investigated molecules encapsulated in macrocyclic host macrocyclic host are significantly different from those observed in bulk solution. These significant changes in spectroscopic behaviours were attributed to the formation of stable 1:1 and/or 1:2 inclusion complexes. The effect of macrocyclic host complexation on TICT and ESIPT photochemistry has been interpreted in terms of the protection of the embedded organic molecule in host cavity from the neighbouring water molecules (reduction of hydrogen bonds upon encapsulation), size and shape of macrocyclic host, orientation of the guest molecule in micropockets, restriction on molecular motion and the reduced polarity effect introduced by cavities as compared to the aqueous phase [33,34,35,36,37].

However, important issues on the nature and mechanisms of host guest interactions remain open and require clarification. Herein, we focus on the deepening the knowledge of the nature and mechanisms of interactions between two selected methyl benzoate derivatives (methyl *o*-methoxy *p*-methylaminobenzoate–**I**, methyl *o*-hydroxy *p*-methylaminobenzoate–**II** (Scheme 1B))-showing locally-excited (**I** and **II**) and ESIPT fluorescence (**II**)-and biological cavity (BSA). In particular, it is compared whether or not the investigated fluorophores show similar spectroscopic and photochemical properties in the presence of biological cavities (BSA) as in the case of chemical nanocages (cyclodextrins (α, β and γ) and cucurbit[7]urils). The interaction of **I** and **II** with the model transport protein BSA is studied using steady-state and state-of-the-art time-resolved spectroscopic techniques, which proved to be helpful in understanding the mechanisms of the processes leading to the formation of a stable small organic molecule-protein complexes (quenching, binding and thermodynamic parameters). Overall, our current (in combination with our previous) studies offer a detailed outlook into the dynamic and structural aspects of chemical (CDs, cucurbit[7]urils) [33,34,35,36,37] and biological (BSA) host–guest interactions. Especially, we investigate the effects exerted by biological and chemical nanocavity on the excited-state intramolecular proton and electron transfer processes that may occur in supramolecular systems.

## 2. Results and Discussion

### 2.1. Steady-State Absorption and Fluorescence Spectra of I and II in the Phosphate Buffer in the Presence of BSA

As mentioned earlier, UV−visible absorption and fluorescence spectroscopy belongs to the most important techniques used to explore small organic molecule−protein interactions. This is due to the fact that the spectroscopic properties of HSA and/or BSA (in fact protein’s fluorophores) are sensitive to the immediate neighbourhood i.e., polarity of their surroundings, as well as composition of the solvation shell. Thus, before undertaking the investigation of the interactions between BSA and two methyl benzoate derivatives (**I** and **II**), it is necessary to recall the spectroscopic behaviour of these two organic fluorophores, BSA, as well as three crucial amino acid residues: tryptophan (Trp), tyrosine (Tyr), and phenylalanine (Phe) (because, as mentioned, spectroscopic properties of the BSA are mainly governed by the absorption/fluorescence of above mentioned residues) [38].

**Figure 1:**
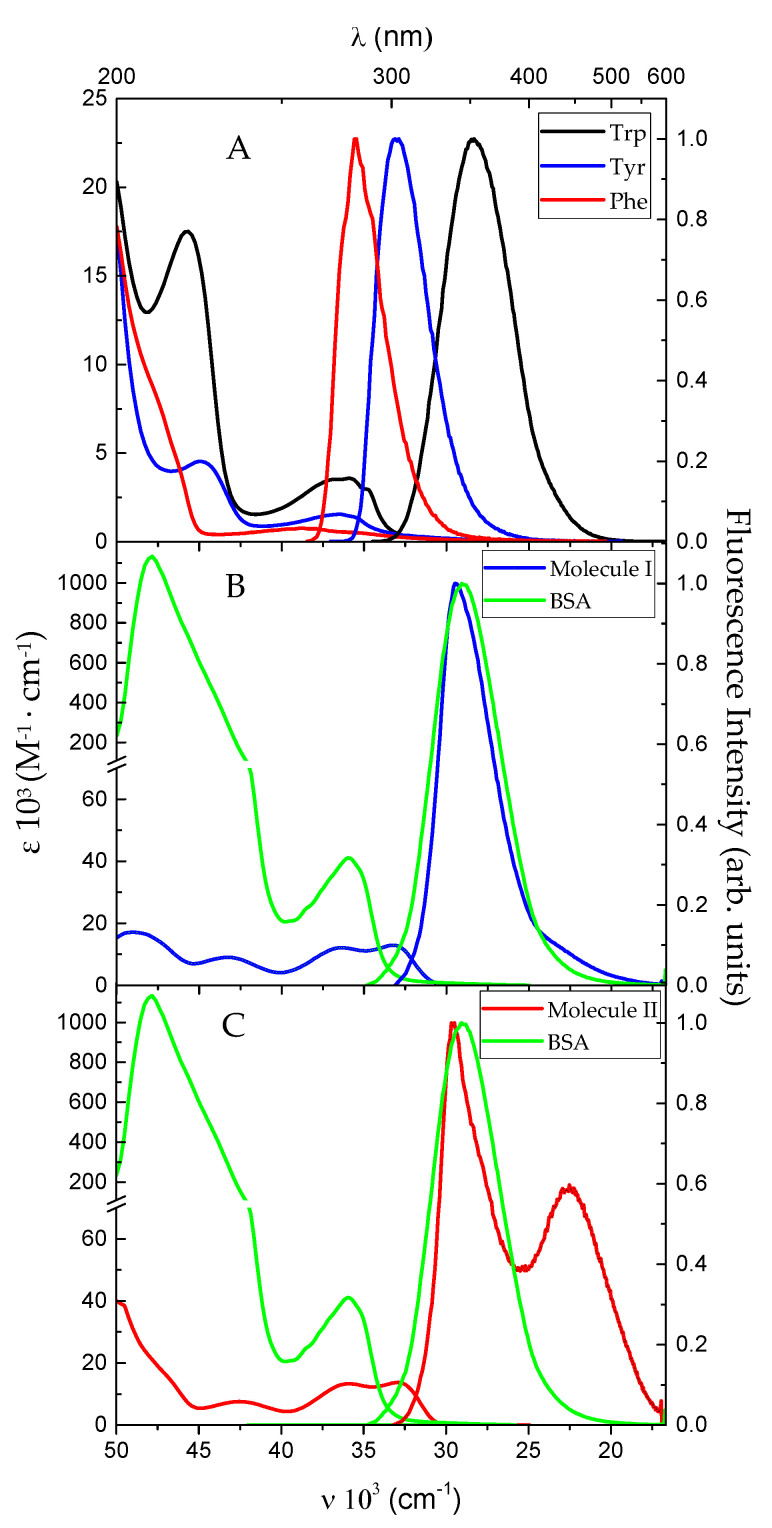
Absorption and emission spectra of the three crucial aromatic amino acids (**A**), BSA (**B**,**C**) and investigated dyes (**B**,**C**) in phosphate buffer solution (pH 7.4). In order to better visualize the absorbance scale (representing the molar absorption coefficient) for BSA and investigated molecules, the ordinate axis has been truncated.

Several spectroscopic investigations were carried out on the **I**-BSA and **II**-BSA systems (and in parallel on the **I**-Trp, **II**-Trp systems) to recognize whether some interactions occurred and to obtain insight into the nature of such interactions. The spectral characteristics of the two investigated methyl benzoate derivatives, BSA and three crucial amino acid residues (Trp, Tyr, Phe) in the phosphate buffer are presented in Figure 1. As can be seen, these residues have distinct absorption and emission wavelengths and differ in the quantum yield (in aqueous medium quantum yields of Trp, Tyr and Phe are near 0.2, 0.14 and 0.03, respectively) [38]. Several experimental and theoretical studies have shown that, proteins emission is dominated by Trp, which absorbs at the longest wavelength ($\lambda_{max}^{abs}\left(Trp\right)$ in phosphate buffer occurs at 280 nm) and displays the largest molar absorption coefficient [38]. Although Tyr quantum yield is similar to that of Trp, emission is often quenched, mostly due to its interactions with the peptide chain or as a result of energy transfer to Trp [38]. As can be seen, Tyr can be excited at wavelengths similar to that of Trp (($\lambda_{max}^{abs}\left(\mathrm{Tyr}\right)$ in phosphate buffer occurs at 275 nm) but emits at a distinctly different wavelength. The wavelength of maximum intensity of the fluorescence band of Tyr is distinctly blue-shifted in comparison to the fluorescence band of Trp ($\lambda_{max}^{fl}\left(\mathrm{Tyr}\right)$ = 303 nm, $\lambda_{max}^{fl}\left(\mathrm{Trp}\right)$ = 351 nm). It is also evident that among the three crucial amino acids, Phe displays the shortest absorption and emission wavelengths. Considering that proteins fluorescence is generally excited at 280 nm or at longer wavelengths, Phe is not excited in the majority of experiments (this holds true also in our case).

In previous reports [28,29,30,31,32], we have shown that the long-wavelength absorption band of the studied fluorophores in neat solvents consists of two electronic transitions $S_{0}\to S_{1}$ and $S_{0}\to S_{2}$ of different character: ππ* and charge-transfer (CT). Moreover, it is well established that molecule **II** dissolved in some media besides the locally-excited (LE) fluorescence exhibits the long-wavelength emission from the excited-state intramolecular proton transfer (ESIPT) state [28,29,30,31,32]. In Figure 1B,C, the long-wavelength absorption and fluorescence spectra of BSA in the phosphate buffer are compared with those of **I** and **II**. As the spectral behaviours of BSA mainly arise due to the presence of two tryptophan residues (Trp-212 (located in hydrophobic subdomain IB) and Trp-134 (located near the surface of the albumin molecule in the second helix of the first domain)), the steady-state absorption and fluorescence spectra of BSA and Trp show similar spectral features ($\lambda_{max}^{abs}\left(\mathrm{Trp}\right)$ = 280 nm versus $\lambda_{max}^{abs}\left(\mathrm{BSA}\right)$ = 278 nm, $\lambda_{max}^{fl}\left(\mathrm{Trp}\right)$ = 351 nm versus $\lambda_{max}^{fl}\left(\mathrm{BSA}\right)$ = 344 nm). The long-wavelength absorption spectrum of BSA overlaps with the broad, long-wavelength absorption band (consisting of two different electronic transitions) of the investigated methyl benzoate derivatives, as well as the short-wavelength, locally-excited fluorescence band of **I** and **II** considerably overlap with that of serum protein.

**Figure 2:**
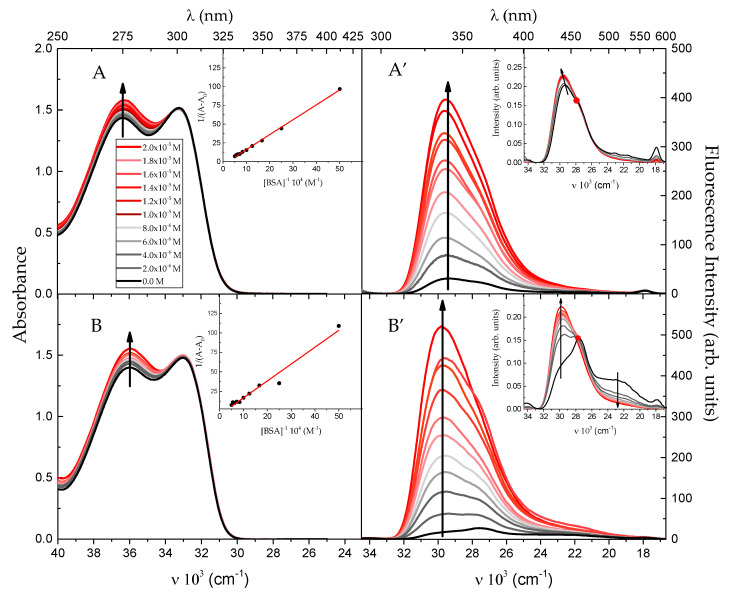
Absorption and fluorescence spectra (λ_exc._ = 300 nm) of **I** (**A**,**A’**), **II** (**B**,**B’**) (c = 5 × 10^−5^ M) in phosphate buffer solutions containing different concentration of BSA (0 M, 2.0 × 10^−6^ M, 4.0 × 10^−6^ M, 6.0 × 10^−6^ M, 8.0 × 10^−6^ M, 1.0 × 10^−5^ M, 1.2 × 10^−5^ M, 1.4 × 10^−5^ M, 1.6 × 10^−5^ M, 1.8 × 10^−5^ M, 2.0 × 10^−5^ M). Insert the ground-state Benesi–Hildebrand dependence for **I** (**A**), **II** (**B**) and the normalized fluorescence spectra of **I** (**A’**) and **II** (**B’**) in phosphate buffer solutions containing different concentration of BSA.

In order to obtain more insight into **I**-BSA and **II**-BSA interaction process, the absorption- and fluorescence-titration experiments were performed with varying concentrations of BSA. Figure 2 shows the LW absorption spectra of **I** and **II** in the phosphate buffer solutions containing different concentrations of BSA. Upon addition of BSA, the molar absorption coefficient of the first long-wavelength absorption band centred at about 275 nm (molecule **I**) and 279 nm (molecule **II**) gradually increases, whereas no significant changes in the second long-wavelength absorption band (centred at 300 nm (**I**) and 305 nm (**II**)) were observed in the presence of BSA. Considering the absorption characteristics of BSA in phosphate buffer solution, this spectral behaviour probably comes from the absorption of BSA molecule. Moreover, an isosbestic point can be clearly seen between these two bands, which indicates the presence of equilibrium between two different species (**I** (or **II**) and the ground-state complex **I**-BSA (or **II**-BSA)).

Addition of BSA to the phosphate buffer solutions of **I** and **II** brings about a strong increase in the fluorescence intensity with a slight shift in the position of the short-wavelength fluorescence band maximum (~4 nm). Moreover, when the protein concentration is increased, an isosbestic point can be clearly seen in normalized fluorescence spectra (we normalized each of the area under the fluorescence curve to unity, in this case, fluorescence spectrum corresponds to the emission of the same number of emitting molecules) points that equilibrium is established also in the excited state for both studied systems (see insert of Figure 2). The appearance of an isosbestic point-like feature in the normalized fluorescence spectra of molecule which is capable of exhibiting ESIPT-type behaviour (molecule **II**) and gradual decrease in the intensity of the ESIPT fluorescence band indicate that the binding between **II** and BSA is specific and involves the hydroxy (-OH) and/or ester (-COOCH_3_) groups, which causes that ESIPT does not appear.

The changes in the absorbance and fluorescence intensity of **I** and **II** in the presence of BSA have been used to create the Benesi−Hildebrand (B−H) plot (see Equation (2a,b)). As can be seen in the insert of Figure 2 and Figure S1 (Supplementary Materials), for both investigated systems, the plots of $1/\left(A-A_{0}\right)$ versus 1/[BSA], as well as $1/\left(I-I_{0}\right)$ versus 1/[BSA]) demonstrate linear dependence, which indicates the formation of well-defined 1:1 complexes with BSA, both in the ground and excited states. The binding constants were found to be (1.9 ± 0.1) × 10^4^ M^−1^ and (2.3 ± 0.1) × 10^4^ M^−1^ in the ground state and (2.1 ± 0.1) × 10^4^ M^−1^ and (2.0 ± 0.1) × 10^4^ M^−1^ in the excited state for **I** and **II**, respectively. These results indicate the strong binding affinity of the investigated dyes to BSA in the ground and excited states.

### 2.2. Time-Resolved Studies of I and II in the Phosphate Buffer in the Presence of BSA

To obtain information on the excited-state dynamics of the methyl benzoate derivative-BSA system in the phosphate buffer, the time-resolved emission spectra (TRES) of the investigated dyes upon the gradual addition of BSA were recorded. Table 1 gives the fluorescence lifetimes and preexponential factors describing the contribution of the i-th fluorescence decay component to the total emission.

We will first discuss the changes in the fluorescence lifetimes of molecule **II**, which is capable of exhibiting ESIPT-type behaviour, in the presence of BSA in the phosphate buffer medium. As expected, fluorescence decay profile of **II** recorded in the absence of BSA consists of a slow decay *τ*_2_ = 0.8 ns as a major component (76%) with a small contribution of a fast decay component (*τ*_1_ = 200 ps, *A*_1_ = 24%). According to our previous works [35,37], we can state that the slow component can be assigned to the emission from ESIPT state, whereas the decay time of the fast component originates from the emission of LE state.

**Figure 3:**
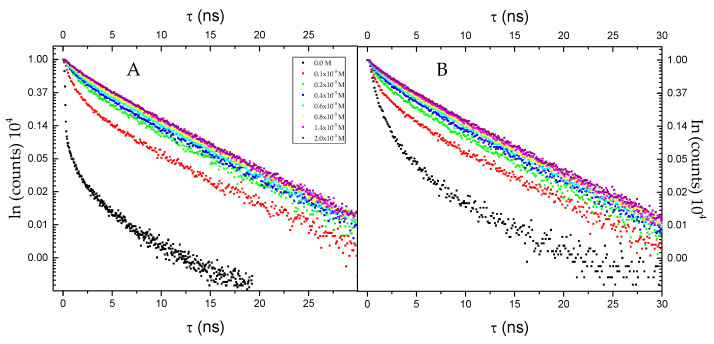
Fluorescence decay profiles of **I** (**A**) and **II** (**B**) in absence and different concentration of BSA (0.0 M, 0.1 × 10^−5^ M, 0.2 × 10^−5^ M, 0.4 × 10^−5^ M, 0.6 × 10^−5^ M, 0.8 × 10^−5^ M, 1.4 × 10^−5^ M, 2.0 × 10^−5^ M).

The fluorescence kinetics of **II** in the presence of BSA are of the most complex nature (see Table 1 and Figure 3). In the presence of BSA at low concentration (10^−6^–6 × 10^−6^ M), the decay fits to three-exponential function with time constants of $\tau_{1}$~200 ps, $\tau_{2}$~1 ns and $\tau_{3}$~5.7 ns, whereas at the highest concentration (8 × 10^−6^–2 × 10^−5^ M), fluorescence decays can be reasonably well fitted by monoexponential function ($\tau_{3}$~5.7 ns) (see Table 1 and Figure 3). Through the individual form of the time constants of **II** (LE and ESIPT) remain almost unchanged, their relative preexponential factors are distinctly modified upon interactions with BSA. The preexponential factors associated with the LE an ESIPT emission ($A_{1}$ and $A_{2}$) are found to be decreased from 24% and 76% to 1% and 6%, respectively, whereas $A_{3}$ factor presents opposite behaviour. These results clearly indicate that the short components ($\tau_{1}$ and $\tau_{2}$) correspond to the unbound forms (LE and ESIPT), while the long component $\tau_{3}$ corresponds to the bound form (**II**-BSA). Moreover, this binding results in the absence of the intramolecular proton transfer process in the excited state, confirming the involvement of the hydroxy (-OH) and/or ester (-COOCH_3_) groups in specific interactions.

For the molecule **I** in the phosphate buffer medium, the fluorescence decay profile can be satisfactorily fitted by monoexponential function with the fluorescence decay time $\tau_{1}$~200 ps (Table 1), in the presence of BSA at low concentration (10^−6^–6 × 10^−6^ M), the sum of two exponential functions was required, whereas at the highest concentration (8 × 10^−6^–2 × 10^−5^ M), again only one emissive centre was observed ($\tau_{3}$~5.7 ns) (Figure 3). In the presence of low concentration of BSA (10^−6^ M), fluorescence kinetics exhibits a slow decay component $\tau_{3}$ as a major component (93%), which is close to the average lifetime of two Trp moieties [21], with a small contribution of fast decay component ($\tau_{1}$~200 ps). It should be also clearly state that, the values of fast and slow decay components ($\tau_{1}$ and $\tau_{3}$) remain almost unchanged ($\tau_{1}$ slightly increases) with increasing BSA concentration. Conversely, the preexponential factor *A*_1_ corresponding to the decay of LE form of the molecule **I** decreases with increasing BSA concentration, whereas *A*_3_ factor presents opposite behaviour. These findings indicate that the possibility of the emission from LE state is limited due to the presence of specific interactions between **I** and BSA (formation of **I**-BSA complex).

**Figure 4:**
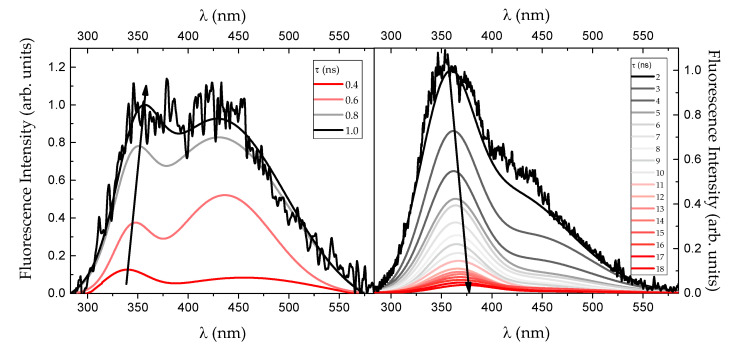
Time-resolved emission spectra (λ_exc._ = 300 nm) of **II** in phosphate buffer solution with BSA (c_BSA_ = 10^−6^ M) at various times after excitation.

**Figure 5:**
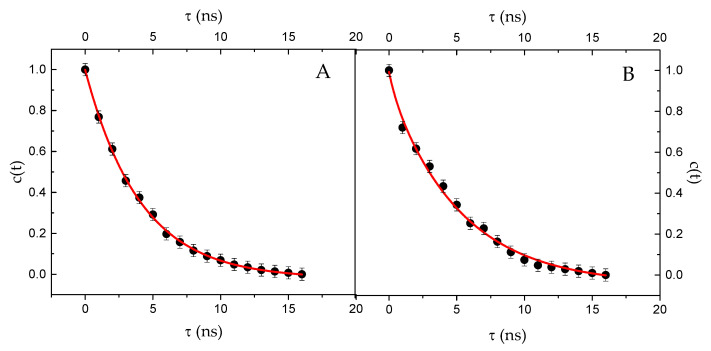
Temporal behaviour of *c*(*t*) function for the short-wavelength fluorescence band of **I** (**A**) and **II** (**B**) in phosphate buffer solution with BSA (c_BSA_ = 10^−6^ M).

In our previous paper, the mechanism of supramolecular solvation dynamics of the methyl benzoate derivatives in aqueous solution with CB[7] has been investigated according to the procedure described by Maroncelli and Fleming [37,39,40]. We have shown that solvation dynamics which occurs in subpicosecond time scale in neat solvents of different polarities is slowed down significantly when organic molecule is relocated to CB[7] cavity. In order to determine and compare solvation dynamics in selected chemical and biological nanocavities (i.e., CB[7]) versus BSA), the time-dependent fluorescence Stokes’ shifts (determined from the emission spectra registered at various times after excitation (see Figure 4), were used to create the normalized solvation correlation function *c*(*t*) (see Equation (7)). Figure 5 presents the temporal behaviour of *c*(*t*) function for **I** and **II** bound to BSA. For both molecules, decay of *c*(*t*) is well fitted by the sum of two exponential functions (see Equation (8)) with a fast component τ_1(solv.)_ (**I**) = 430 ps, *τ*_1(solv.)_ (**II**) = 520 ps (*A*_1(solv.)_ (**I**) = 2%, *A*_1(solv.)_(**II**) = 3%) and a slow component of τ_2(solv.)_ (**I**) = 4.07 ns, *τ*_2(solv.)_ (**II**) = 5.25 ns (*A*_1(solv.)_ (**I**)= 98%, *A*_1(solv.)_ (**II**) = 97%), with an average solvation time 〈*τ*_(solv.)_ (**I**)〉 = 4.06 ns, and 〈*τ*_(solv.)_(**II**)〉 = 5.24 ns.

In order to explain the presence of the nanosecond component of the solvation dynamics observed in our studies, it is necessary to assume the restriction on the movement of the biological water molecules in the vicinity of the protein. Similar behaviour has been reported previously and explained as a result of three main causes: (i) in bulk, the movement of water is cooperative and involves an extensive network of hydrogen bonds, (ii) in the case of protein-bound water, the hydrogen bond network is significantly disturbed, (iii) the different solvation times of biological water molecules may correspond to different parts of albumin [41,42]. Thus, the present investigation clearly shows that the solvation dynamics of **I** and **II** in aqueous solution with CB[7] is different from that in biological nanocavities (BSA). The average solvation time in methyl benzoate derivative-BSA system is found to be about one order of magnitude longer than that in **I**-CB[7] (or **II**-CB[7]) system. This indicates that at BSA hydrophobic interior, where investigated organic molecule is buried, the water molecules are more strongly confined than in the CB[7] cavity and therefore show slower solvation dynamics.

### 2.3. Effect of Trp on the Steady-State and Time-Resolved Spectroscopic Behaviour of I and II

**Figure 6:**
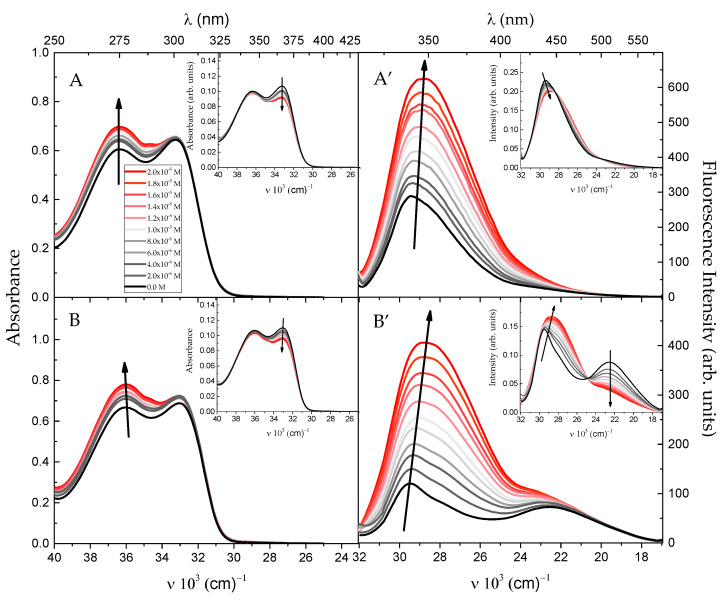
The long-wavelength absorption and fluorescence spectra (λ_exc._ = 300 nm) of **I** (**A**,**A’**) and **II** (**B**,**B’**) (c = 5 × 10^−5^ M) in phosphate buffer solutions containing different concentration of Trp (0 M, 2.0 × 10^−6^ M, 4.0 × 10^−6^ M, 6.0 × 10^−6^ M, 8.0 × 10^−6^ M, 1.0 × 10^−5^ M, 1.2 × 10^−5^ M, 1.4 × 10^−5^ M, 1.6 × 10^−5^ M, 1.8 × 10^−5^ M, 2.0 × 10^−5^ M). In order to better visualize the changes in the absorption (in the long-wavelength (LW) region) and emission spectra, the normalized spectra (by scaling the area under the spectrum to be equal to unity) are presented in insert.

In order to understand the role of Trp residue of BSA in the **I**-BSA and **II**-BSA interaction, it is useful to also study the interactions between molecules under study and the free Trp amino acid. As seen in Figure 6, the steady-state absorption and fluorescence spectra of buffered solutions of **I** and **II** are also affected by the presence of tryptophan molecules. For both fluorophores, with the gradual addition of Trp, the molar absorption coefficient of the first long-wavelength absorption band increases without change in spectral distribution of the second LW absorption band. Moreover, the occurrence of an isosbestic point in the titration experiment suggests that the equilibrium is established between methyl benzoate derivative (**I** or **II**) and the methyl benzoate derivative-Trp complex in the ground state. The excited-state spectral behaviours of **I**-Trp and **II**-Trp systems (e.g., increase in the fluorescence intensity and presence of an isosbestic point in the steady-state area normalized emission spectra) are similar to those in **I**-BSA and **II**-BSA systems suggesting that methyl benzoate derivative-Trp complex is also formed in the excited state. It is also evident from the normalized fluorescence spectra that for fluorophore **II**, upon increasing the Trp concentration, the intensity of the ESIPT fluorescence band decreases and simultaneously the short-wavelength (LE) emission band increases. The described experiment indicates (as was the case with **II**-BSA system) the involvement of the -OH and/or -COOCH_3_) substituents in specific interactions between **II** and Trp. Here, the same absorption- and fluorescence-titration experiments were performed for **I**-Tyr, **II**-Tyr, **I**-Phe and **II**-Phe systems. For these systems, the absorption and fluorescence spectra of **I** and **II** in phosphate buffer solution containing different concentration of two amino acid residues (Tyr and Phe) were practically unchanged, which indicates that probably no specific interaction with Tyr and Phe occurs.

As can be seen in Figure S2, for both studied systems, the plots of $1/\left(A-A_{0}\right)$ versus 1/[Trp] and $1/\left(I-I_{0}\right)$ versus 1/[Tryp]  show linear dependence over the whole range of studied Trp concentration, confirming the formation of complexes with 1:1 stoichiometry between methyl benzoate derivatives (**I** and **II**) and Trp in the ground and excited states. The binding constants were found to be (2.1 ± 0.1) × 10^4^ M^−1^ and (2.6 ± 0.1) × 10^4^ M^−1^ in the ground state and (1.9 ± 0.1) × 10^4^ M^−1^ and (2.6 ± 0.1) × 10^4^ M^−1^ in the excited state for **I** and **II**, respectively, which is similar to the previously calculated values for **I**−BSA and **II**-BSA complexes. This behaviour may indicate that mainly the Trp residue of BSA is involved in process of I-BSA and II-BSA specific interactions.

As can be seen in Table 2, the fluorescence kinetics of the investigated molecules in the presence of Trp is similar to that in **I**-BSA and **II**-BSA systems ((i) decay components ($\tau_{1},\;\tau_{2}$ and $\tau_{3}$) remain almost unchanged; (ii) the preexponential factors *A*_1_ and *A*_2_ corresponding to decay of LE and ESIPT (molecule **II**) emission decrease with increasing Trp concentration, whereas *A*_3_ factor (corresponding to the decay of bound form (**I**-Trp and **II**-Trp)) presents opposite behaviour) confirming the formation of complex between methyl benzoate derivatives (**I** and **II**) and Trp.

**Scheme 2:**
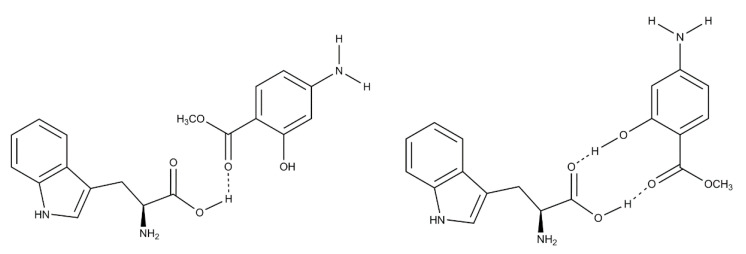
Two of the possible structures of complexes formed between **II** and Trp molecule.

The presented above findings suggest that mainly Trp residue of BSA is involved in the process of specific interaction between **I** (or **II**) and BSA. Considering possible location of Trp in BSA, it can be concluded that the molecules **I** and **II** enter the hydrophobic cleft of the IB subdomain (location of the Trp-212 molecule) or near the surface of the albumin molecule in the second helix of the first domain (location of the Trp-134 molecule) and form specific hydrogen bonds with Trp-212 and/or Trp-134, thereby causing static fluorescence quenching of BSA (Trp). Moreover, as the investigated fluorophores have the chemically active substituents which are likely sites for intermolecular hydrogen bonding with Trp, Scheme 2 presents the most probable structures of complexes formed between **II** and Trp molecule. As can be seen, the Trp residue was attached to the investigated molecules only at the sites considered important for hydrogen bond formation. It is obvious that actual situation in **I**-BSA, **II**-BSA, **I**-Trp and **II**-Trp systems is not simple, but the considered model can give a first insight into the specific Trp-methyl benzoate derivative interactions. In future works, separate molecular docking studies will be used to confirm the most probable binding site with BSA.

### 2.4. Effect of I and II on the Steady-State and Time-Resolved Spectroscopic Behaviour of BSA and Trp

**Figure 7:**
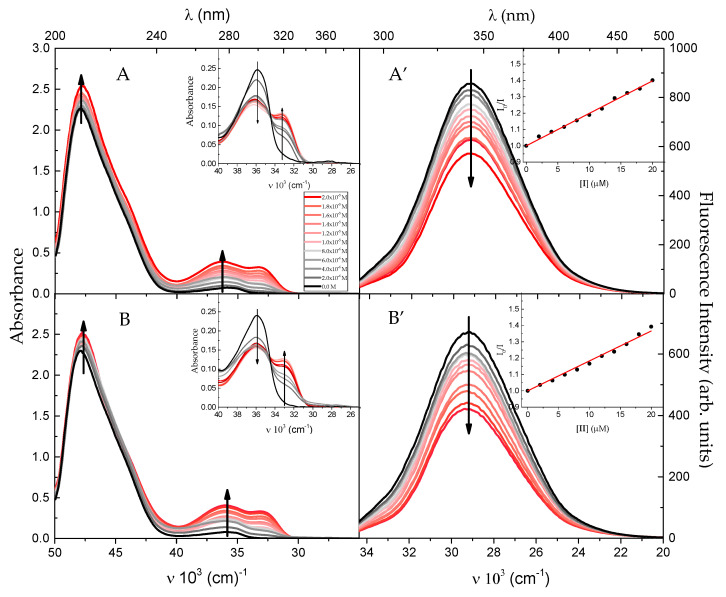
Absorption and fluorescence spectra (λ_exc._ = 280 nm) of BSA (c = 2 × 10^−6^ M) in phosphate buffer solutions containing different concentration of **I** (**A**,**A’**) and **II** (**B**,**B’**) (0 M, 2.0 × 10^−6^ M, 4.0 × 10^−6^ M, 6.0 × 10^−6^ M, 8.0 × 10^−6^ M, 1.0 × 10^−5^ M, 1.2 × 10^−5^ M, 1.4 × 10^−5^ M, 1.6 × 10^−5^ M, 1.8 × 10^−5^ M, 2.0 × 10^−5^ M). The normalized LW absorption bands (by scaling the area under the spectrum to be equal to unity) are presented in insert of (**A**,**B**). The insert in (**A’**,**B’**) shows a graphical presentation of the relationship between I_0_/I versus (I) (**A’**) and (II) (**B’**).

In order to clarify the interaction process between two methyl benzoate derivatives and BSA and Trp, we have also carried out a series of the steady-state absorption and fluorescence studies of BSA and Trp in the presence of **I** and **II**. To begin with, Figure 7 and Figure S3 show the changes observed in the absorption (A,B) and emission (A’,B’) spectra of BSA (Figure 7) and Trp (Figure S3) in the phosphate buffer upon the gradual addition of **I** (A,A’) and **II** (B,B’). In order to better visualize the changes in the absorption spectra in the long-wavelength (LW) region (250–340 nm), the normalized LW absorption bands (by scaling the area under the spectrum to be equal to unity) are presented in insert of Figure 7 and Figure S3. For both investigated systems (BSA-investigated molecules and Trp-investigated molecules), upon the gradual addition of the methyl benzoate derivative to the aqueous buffer solution of BSA (or Trp), the absorbance of the short-wavelength (centred at about 210 nm (BSA-**I** and BSA-**II**) and 218 nm (Trp-**I** and Trp-**II**)) and long-wavelength (centred at about 280 nm (BSA-**I** and BSA-**II**) and 275 nm (Trp-**I** and Trp-**II**)) bands gradually increases and simultaneously a new absorption band centred at around 310 nm appears. An isosbestic point in normalized LW absorption bands can be clearly seen between these two bands (see insert of Figure 7 and Figure S3), which confirms formation of the ground-state complex between BSA or Trp and both investigated dyes.

The interactions of BSA and Trp with two studied dyes were also investigated by the fluorescence quenching spectroscopy. In both cases (BSA and Trp), the addition of methyl benzoate derivative to a solution of BSA (or Trp) quenches significantly the single, broad fluorescence band centred at about 340 nm (BSA) and 352 nm (Trp) (registered upon excitation at 280 nm) without significant changes in the shape of the fluorescence spectrum (see Figure 7 and Figure S3). There is no shift in the wavelength of maximum intensity of BSA and Trp when it was quenched by **I** and **II**.

Using data obtained from the absorption and fluorescence titration experiments (BSA-investigated molecules and Trp-investigated molecules), the Benesi−Hildebrand plots were constructed (Figure S4). Since the plots of $1/\left(A-A_{0}\right)$ versus 1/[I] (1/[II]) and $1/\left(I-I_{0}\right)$ versus 1/[I] (1/[II]) produce a straight line for both systems with a good correlation coefficient (r > 0.95), the stoichiometry of the formed complexes between BSA (Trp) and methyl benzoate derivatives in the ground and excited states is 1:1. The binding constants were found to be (2.1 ± 0.1) × 10^4^ M^−1^ and (2.4 ± 0.1) × 10^4^ M^−1^ in the ground state and (2.0 ± 0.1) × 10^4^ M^−1^ and (2.7 ± 0.1) × 10^4^ M^−1^ in the excited state, for BSA-**I** and BSA-**II**, respectively and (2.0 ± 0.1) × 10^4^ M^−1^ and (2.2 ± 0.1) × 10^4^ M^−1^ in the ground state and (1.8 ± 0.1) × 10^4^ M^−1^ and (2.4 ± 0.1) × 10^4^ M^−1^ in the excited state, for Trp-**I** and Trp-**II**, respectively. It is clearly seen that they are in a good agreement with data presented in previous section (Section 2.1). The binding constant values determined on the basis of the two independent titration experiments differ by about 10%.

In order to confirm the existence of the ground and excited states complexes (BSA-**I** and BSA-**II**, as well as Trp-**I** and Trp-**II**) and the 1:1 stoichiometry of the complexes, the results of the steady-state spectroscopic measurements are analysed using the Job’s plot technique (continuous variation method) [43]. Job plots were generated by plotting the difference in absorbance (ground state complex) or fluorescence intensity (excited state complex) versus the mole fraction of the investigated dye (R) in the used solutions (Figure S5). The curve maximum at about 0.5 supports the previously mentioned 1:1 stoichiometry in the ground and excited states i.e., the number of **I** (or **II**) molecules binding to BSA and Trp is close to unity.

#### 2.4.1. Mechanism of the Observed Fluorescence Quenching

It is well known that the progressive fluorescence quenching can be explained by three main reasons: static quenching (nonfluorescent complex is formed between fluorophore and quencher), dynamic (resulting from collisional encounters between fluorophore and quencher) and quenching both by collisions and complex formation [38]. In order to determine the extent and mechanism of the fluorescence quenching of BSA by **I** and **II** molecules, the results of spectroscopic measurements are analysed using Stern-Volmer equation (see Equation (3)). As can be seen in insert of Figure 7, the Stern-Volmer plot ($I_{0}/I$ versus [**I**] and [**II**]) at 298 K is linear for both systems, which indicates that only one type of quenching occurs (static or dynamic). The K_SV_ values obtained from linear plots are listed in Table 3. From the K_SV_ values obtained for both of the systems at room temperature (298 K), it is evident that the molecule **II** has only a slightly stronger binding affinity for BSA in comparison to **I** (the ratio of the corresponding Stern−Volmer quenching constant, *K_SV_* (**I**)/*K_SV_*(**II**), is about 1.05).

**Figure 8:**
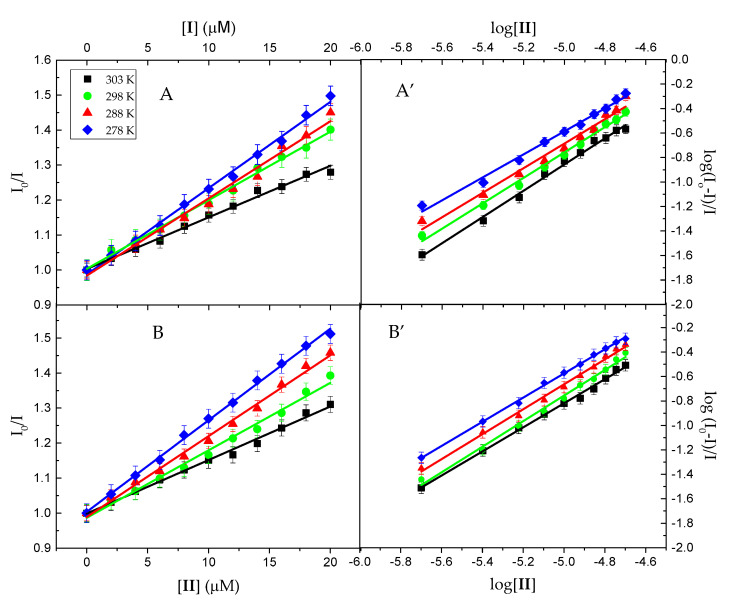
Stern-Volmer (**A**,**B**) and log(I_0_-I)/I versus log(molecule) (**A’**,**B’**) plots for quenching of BSA by **I** (**A**,**A’**) and **II** (**B**,**B’**) at different temperatures.

Because character of fluorescence quenching (static versus dynamic) can be differentiated by their different dependence on temperature (higher temperatures result in faster diffusion and dissociation of weakly bound complexes), temperature-dependent fluorescence spectral measurements were conducted at four different temperatures, and data are analysed using Stern−Volmer relation. Plots of $I_{0}/I$ versus [**I**] and [**II]** at four temperatures are shown in Figure 8. Analysing the K_SV_ values (obtained at different temperatures) assembled in Table 3, in connection with the graphical presentation in Figure 8, it is evident that the quenching mechanism of the investigated systems is sensitive to temperature. As can be seen, upon increasing the temperature, K_SV_ value decreases, which indicates that the quenching process is static i.e., complex is formed between **I** (or **II**) and BSA.

Static character of fluorescence quenching is also confirmed by temperature-dependent Benesi–Hildebrand plot experiment. The plots of 1/(*I* − *I*_0_) follow a linear dependence on 1/[**I**] (1/[**II**]) for four investigated temperatures (278–303 K). This finding indicates 1:1 complex formation between studied compounds and BSA at all temperatures. As seen in Table 3, the increase of the solute temperature from 278 to 303 K results also in the decrease of binding constant (*K_BH_*) value by a factor of 2 (for BSA-**I** system) and 1.5 (for BSA-**II**), which confirms the occurrence of static quenching mechanism - higher temperatures results in dissociation of weakly bound complexes.

It is well-known that the analysis of fluorescence kinetics is the most definite method to distinguish the character of fluorescence quenching (static versus dynamic) [38]. For static quenching, the fluorescence lifetime of the investigated molecule remains unchanged in the presence of a quencher, while for dynamic quenching $\tau_{0}/\tau \neq 1$. In order to further confirm the quenching mechanism, time-resolved emission spectra of BSA upon gradual addition of **I** and **II** were recorded. The lifetime parameters presented in Table 4 show that fluorescence decays consist of a fast (~1 ns (10%)) and a slow (~5.6 ns (90%)) components, with an average time ~5.2 ns. The nonexponential kinetic traces of the native BSA has been described previously and most probably they can be assigned to the presence of two Trp moieties at distinct conformational states [21]. It is clearly seen that the average lifetime of BSA is practically unchanged in the presence of different concentrations of **I** and **II**, which confirms our previous conclusions drawn from the analysis of the steady-state spectroscopic measurements described above i.e., static quenching mechanism between BSA and **I** (or **II**). Moreover, the high value of the quenching rate constant at room temperature (*k_q_*(**I**) = *K_SV_*(**I**)/ *<τ*_0_*>* = 3.6 × 10^12^ M^−1^ s^−1^, *k_q_*(**II**) = *K_SV_*(**II**)/ <*τ*_0_> = 3.4 × 10^12^ M^−1^ s^−1^), which is much higher than typical diffusion-controlled quenching rate constants (2 × 10^10^ M^−^^1^ s^−^^1^) excludes dynamic character of the quenching process (diffusion controlled reaction).

#### 2.4.2. Thermodynamic Parameters

To support the above conclusions i.e., the number of binding sites per protein molecule (*n*) and binding constant value, we also analysed the fluorescence quenching data using Scatchard equation (Equation (4)). As seen in Figure 8, the linear dependence between $log\left(\frac{I_{0}-I}{I}\right)$ and log[I] (and log[II]) is obtained at four temperatures studied. The slope and the intercept values were therefore obtainable and used to calculate *n* and $K_{b}$. The results are assembled in Table 3. The calculated, temperature-dependent $K_{b}$ values confirm that the fluorescence quenching has a static character, in agreement with the expectation that the binding constant for the static quenching decreases with increasing temperature. Moreover, $K_{b}$ values correlate well with the alternatively calculated binding constant ($K_{BH}^{}$) using Benesi–Hildebrand relation (see Table 3). Finally, *n* values (found to be about 1 for both studied systems) further confirm the 1:1 stoichiometry of the complexes.

In order to understand the role of different types of non-covalent interactions, namely, van der Waals forces, multiple hydrogen-bonding, hydrophobic, and electrostatic interactions, in the binding of **I** (or **II**) with BSA, related thermodynamic characteristics were estimated by using the van’t Hoff equation (Equation (5)). The values of ΔH and ΔS are determined from the slope and intercept of the plot of $\ln K_{b}$ versus 1/T for four different temperatures and presented in Table 3. The negative free energy change (ΔG) value determined for both systems indicates that the interaction of BSA with two studied dyes is a spontaneous reaction. Moreover, from the magnitude and the sign of the ΔS and ΔH thermodynamic parameters (ΔS<0, ΔH<0 and |ΔH|≫|ΔS|), it can also be deduced that: (i) BSA-**I** and BSA-**II** interaction is an exothermic reaction (negative enthalpy change, ΔH<0); (ii) reaction is more enthalpy-driven (negative entropy change (ΔS<0) is balanced by highly negative enthalpy change (ΔH<0) i.e., |ΔH|≫|ΔS|; (iii) hydrogen bonding and weak van der Waals forces play a crucial role in methyl benzoate derivative-BSA interactions.

## 3. Materials and Methods

### 3.1. Reagents and Materials

Bovine serum albumin (BSA, min. 98%, from Sigma-Aldrich, St. Louis, MO, USA) and tryptophan (Trp, 98%, from Sigma-Aldrich), tyrosine (Tyr, 98%, from Sigma-Aldrich), phenylalanine (Phe, 98%, from Sigma-Aldrich) and phosphate buffer solution (pH 7.4) were used as received. The methyl *o*-methoxy *p*-methylaminobenzoate–**I** and methyl *o*-hydroxy *p*-methylaminobenzoate-**II** have been synthesized and purified by Gormin [44,45,46]. The purity of dyes was controlled by thin layer chromatography.

### 3.2. Apparatus and Methods

#### 3.2.1. Steady-State and Time-Resolved Measurements

Absorption and fluorescence spectra were recorded using, respectively, a Shimadzu UV-2401 PC spectrophotometer and Shimadzu RF-5301 PC spectrofluorometer. The emission spectra were corrected for the spectral response of the photomultiplier (Hamamatsu R-928) and monochromator pass, but not for reabsorption which was negligible in these samples. Time-resolved emission spectra were taken with the steak camera (C4334-01 Hamamatsu) and 2501 S spectrograph system (Bruker Optics). Solid state Nd: YAG laser (PL 2143A/SS EKSPLA) and optical parametric generator (PG 401/SH EKSPLA) were used as the picosecond excitation light pulses source [47].

#### 3.2.2. Determination of Stoichiometry and Binding Constants

The determination of the ground (*g*) and excited (*e*) state binding constant and stoichiometry of the formed complex

(1) $$X+Y\overset{K_{1}^{e\;\left(g\right)}}{↔}\left(X:Y\right)+Y\overset{K_{2}^{e\;\left(g\right)}}{↔}\left(X:Y_{2}\right)$$

can be determined by analysing the changes in the absorbance and/or fluorescence intensity of the molecule *X* in the presence of *Y*. From the fluorescence (absorption) titration data, the binding constant was determined by Benesi−Hildebrand equation [48]:

(2a) $$\frac{1}{A-A_{0}}=\frac{1}{K_{n}^{g}\left(A_{1}-A_{0}\right)}\;\cdot \;\frac{1}{{\left[Y\right]}^{n}}+\frac{1}{A_{1}-A_{0}}$$

(2b) $$\frac{1}{I-I_{0}}=\frac{1}{K_{n}^{e}\left(I_{1}-I_{0}\right)}\;\cdot \;\frac{1}{{\left[Y\right]}^{n}}+\frac{1}{I_{1}-I_{0}}$$

In Equation (2), *n* is binding stoichiometry, *I*_0_ (*A*_0_), *I* (*A*) and *I*_1_ (*A*_1_) are the fluorescence intensities (or absorbances) of the molecule *X*, respectively in the absence of *Y*, intermediate and at infinite concentration of *Y*. Thus, the $1/\left(I-I_{0}\right)$ (or $1/\left(A-A_{0}\right)$) should follow a linear dependence on $1/{\left[Y\right]}^{n}$ for the correct stoichiometry (*n*).

The binding stoichiometry was also determined from Job’s method (continuous variation method) [43]. Job plots were constructed by plotting the difference in absorbance and/or fluorescence intensity (Δ*A* (Δ*I*)) of *X* observed in the presence of *Y* against the mole fraction of the dye *X* (x_x_). The value of mole fraction of *X* at the maximum deviation yields the stoichiometry of the formed complex i.e., stoichiometry of *X*:*Y* complex is 1:2 if x_x_ = 0.33; 1:1 if x_x_ = 0.5; 2:1 if x_x_ = 0.66 etc.

#### 3.2.3. Analysis of Fluorescence Quenching Data

In order to determine the extent and mechanism of the fluorescence quenching, the results of spectroscopic measurements are analysed using Stern–Volmer equation [38]:

(3) $$\frac{I_{0}}{I}=1+k_{q}\tau_{0}\left[Q\right]=1+K_{SV}\left[Q\right]$$

where $I_{0}$ and I are the fluorescence intensities in the absence and presence of different concentration of quencher ([Q]), respectively. $k_{q}$, $\tau_{0}$ and $K_{SV}$ are bimolecular quenching constant, average fluorescence lifetime of the molecule in the absence of quencher and Stern-Volmer quenching constant, respectively.

Additionally, using fluorescence quenching data, Scatchard equation (Equation (4)) was employed to estimate the binding constant ($K_{b}$) and the number of binding sites per protein molecule (*n*) [49,50,51]:

(4) $$log\frac{I_{0}-I}{I}=\log K_{b}+n\;\log\left[Q\right]$$

#### 3.2.4. Analysis of Thermodynamic Parameters

The related thermodynamic characteristics were determined from the van’t Hoff equation [52]:

(5) $$\ln K_{b}=-\frac{\Delta H}{RT}+\frac{\Delta S}{R}$$

where $K_{b}$ is the binding constant at temperature *T* and *R* is universal gas constant, ΔH and ΔS are the changes in enthalpy and entropy of the binding process. The Gibb’s free energy change (ΔG) during the binding processes was calculated by using the equation:

(6) ΔG=ΔH−T ΔS

at each corresponding temperature.

#### 3.2.5. Analysis of Supramolecular Solvation Dynamics

The supramolecular solvation dynamics was analysed by the decay of the solvation correlation function *c*(*t*), defined by Maroncelli and Fleming as [39,40]:

(7) $$c\left(t\right)=\;\frac{\tilde{\upsilon }\left(t\right)-\;\tilde{\upsilon }\left(\infty \right)}{\tilde{\upsilon }\left(0\right)-\;\tilde{\upsilon }\left(\infty \right)}$$

where $\tilde{\upsilon }\left(t\right)$, $\tilde{\upsilon }\left(0\right)$, and $\tilde{\upsilon }\left(\infty \right)$ are the wavenumbers of the fluorescence band maximum at times t, 0, and ∞. The decay of the solvation correlation function, c(t), was fitted to an exponential decay:

(8) $$c\left(t\right)=\;\overset{N}{\underset{i=1}{\sum }}A_{i}e^{-t/\tau_{i}}.$$

## 4. Conclusions

In the present work, an attempt was made to understand the nature and mechanisms of interactions between two selected methyl benzoate derivatives (**I** and **II**) and bovine serum albumin. In order to clarify the role of Trp residue of BSA in the **I**-BSA and **II**-BSA interaction phenomenon, **I**-Trp and **II**-Trp systems were also investigated. Experimental results show that the investigated interactions (with both BSA and Trp) are mainly based on the ground- and excited-state complex formation processes. Both molecules form a stable complex with BSA and Trp (with 1:1 stoichiometry) in the ground and excited states. The binding constant was in the order of 10^4^ M^−1^. Moreover, our experimental data clearly indicate that mainly Trp residue of BSA is involved in the process of specific interaction between **I** (or **II**) and BSA.

The fluorescence quenching studies along with time-resolved fluorescence measurements show that the binding of the **I** and **II** causes fluorescence quenching of BSA through a static mechanism, revealing a 1:1 interaction. The binding reaction is associated with the negative Δ*S*, Δ*H* and Δ*G* which implies that the hydrogen bonding and van der Waals forces play major roles in stabilizing the complex. Moreover, the reaction is spontaneous, exothermic and more enthalpy-driven. Finally, our studies have shown that solvation dynamics, which occurs in subpicosecond time scale in neat solvents of different polarities, is slowed down significantly when organic molecule is transferred to BSA cavity. In future works, separate molecular docking studies will be used to confirm the most probable binding site with BSA.

## Figures and Tables

**Table 1:** Fluorescence lifetimes of **I** and **II** in phosphate buffer solutions containing different concentrations of BSA.

Molecule	BSA Concentration 10^−5^ (M)	*τ*_1_ (ns)	*A*_1_ (%)	*τ*_2_ (ns)	*A*_2_ (%)	*τ*_3_ (ns)	*A*_3_ (%)	r^2^
**I**	0	0.19	100					0.98
	0.1	0.20	7			4.49	93	0.99
	0.2	0.30	3			5.36	97	0.99
	0.4	0.30	2			5.18	98	0.99
	0.6	0.32	1			5.57	99	0.99
	0.8					5.51	100	0.99
	1.4					5.69	100	0.99
	2.0					5.78	100	0.99
**II**	0	0.20	24	0.80	76			0.95
	0.1	0.21	14	1.15	15	5.79	71	0.99
	0.2	0.19	5	1.19	17	5.56	78	0.99
	0.4	0.19	2	1.00	9	5.71	89	0.99
	0.6	0.18	1	1.00	6	5.23	93	0.99
	0.8					5.58	100	0.99
	1.4					5.52	100	0.99
	2.0					5.84	100	0.99

**Table 2:** Fluorescence lifetimes of **I** and **II** in phosphate buffer solutions containing different concentration of Trp.

Molecule	Trp Concentration 10^−5^ (M)	*τ*_1_ (ns)	*A*_1_ (%)	*τ*_2_ (ns)	*A*_2_ (%)	*τ*_3_ (ns)	*A*_3_ (%)	r^2^
**I**	0	0.19	100					0.97
	0.4	0.14	12			3.45	88	0.99
	0.8	0.12	6			3.34	94	0.99
	1.2	0.12	5			3.5	95	0.99
	1.6	0.13	3			3.5	97	0.99
	2.0	0.14	2			3.5	98	0.99
**II**	0	0.20	24	0.80	76			0.95
	0.4	0.13	4	0.65	15	3.46	81	0.98
	0.8	0.11	3	0.87	7	3.71	90	0.99
	1.2	0.16	1	0.61	3	3.49	96	0.96
	1.6					2.90	100	0.98
	2.0					2.98	100	0.98

**Table 3:** Stern–Volmer quenching constant (*K_SV_*), binding constant determined using Benesi–Hildebrand (*K_BH_*) and Scatchard (*K_b_*) methods and thermodynamic parameters (binding number (*n*), changes in enthalpy (Δ*H*), entropy (Δ*S*), and Gibb’s free energy (Δ*G*) of the binding process) at different temperatures.

Molecule	Temp. (K)	*K_SV_* 10^4^ (M^−1^)	*K_BH_* 10^4^ (M^−1^)	*K_b_* 10^4^ (M^−1^)	*n*	Δ*H* (kJ)	Δ*S* (J)	Δ*G* (kJ)
**I**	278	2.4	2.6	3.5	1.07	−25.66	−4.86	−24.308
	288	2.1	2.4	2.7	1.04			−24.260
	298	1.9	2.0	2.1	1.00			−24.211
	303	1.5	1.3	1.3	0.93			−24.187
**II**	278	2.6	3.6	3.5	1.07	−25.12	−2.17	−24.512
	288	2.2	3.0	3.0	1.05			−24.491
	298	1.8	2.7	2.7	1.02			−25.469
	303	1.5	1.8	1.3	0.99			−25.458

**Table 4:** BSA fluorescence lifetimes as a function of **I** and **II** concentration at room temperature.

Molecule	Molecule Concentration 10^−5^ (M)	*τ*_1_ (ns)	*A*_1_ (%)	*τ*_2_ (ns)	*A*_2_ (%)	<*τ*> (ns)	r^2^
**I**	0	0.98	6.6	5.59	93.4	5.28	0.99
	0.4	0.98	7.8	5.70	92.2	5.35	0.99
	0.8	0.93	10.4	5.64	89.6	5.15	0.99
	1.2	0.87	12.8	5.49	87.2	4.89	0.99
	1.6	0.91	10.8	5.55	89.2	5.05	0.99
	2.0	0.91	13.2	5.56	86.8	4.94	0.99
**II**	0	0.98	6.6	5.59	93.4	5.28	0.99
	0.4	0.97	7.2	5.58	92.8	5.25	0.99
	0.8	1.01	10.8	5.68	89.2	5.18	0.99
	1.2	0.96	11.5	5.53	88.5	5.02	0.99
	1.6	0.83	10.4	5.40	89.6	4.93	0.99
	2.0	0.97	9.6	5.70	90.4	5.24	0.99

## Data Availability

Not applicable.

## Supplementary Materials

The following are available online at https://www.mdpi.com/article/10.3390/ijms222111705/s1.
