# Supplementary material for: Insight into Molecular Interactions of Two Methyl Benzoate Derivatives with Bovine Serum Albumin
Source: Int J Mol Sci. 2021 Oct 28;22(21):11705. doi: 10.3390/ijms222111705 (PMC8584066; doi:10.3390/ijms222111705)
Supplement: Supplementary file 1 [file ijms-22-11705-s001.zip › ijms-1424993-supplementary.pdf]

Supplementary Materials:

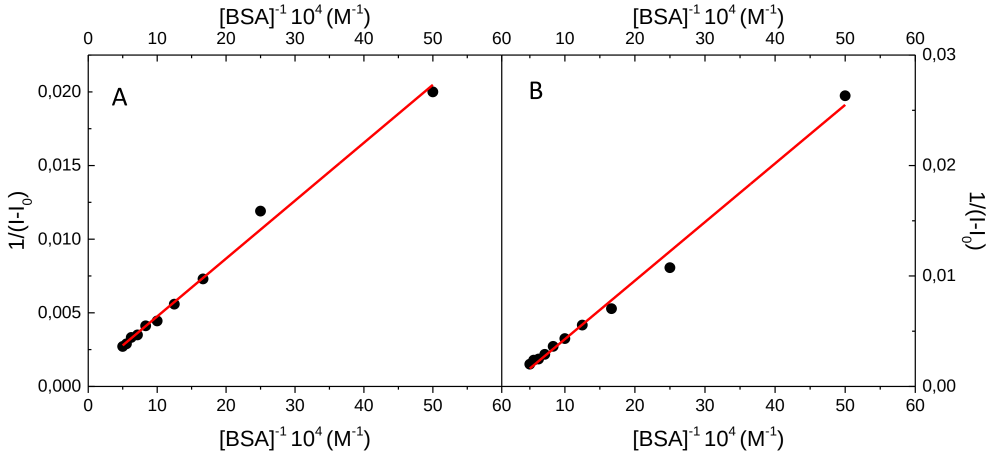

Figure S1. The Benesi-Hildebrand dependence (1/(I-I$_0$) *vs.* [BSA]$^{-1}$) for **I** (**A**) and **II** (**B**) in phosphate buffer solutions containing different concentration of BSA.

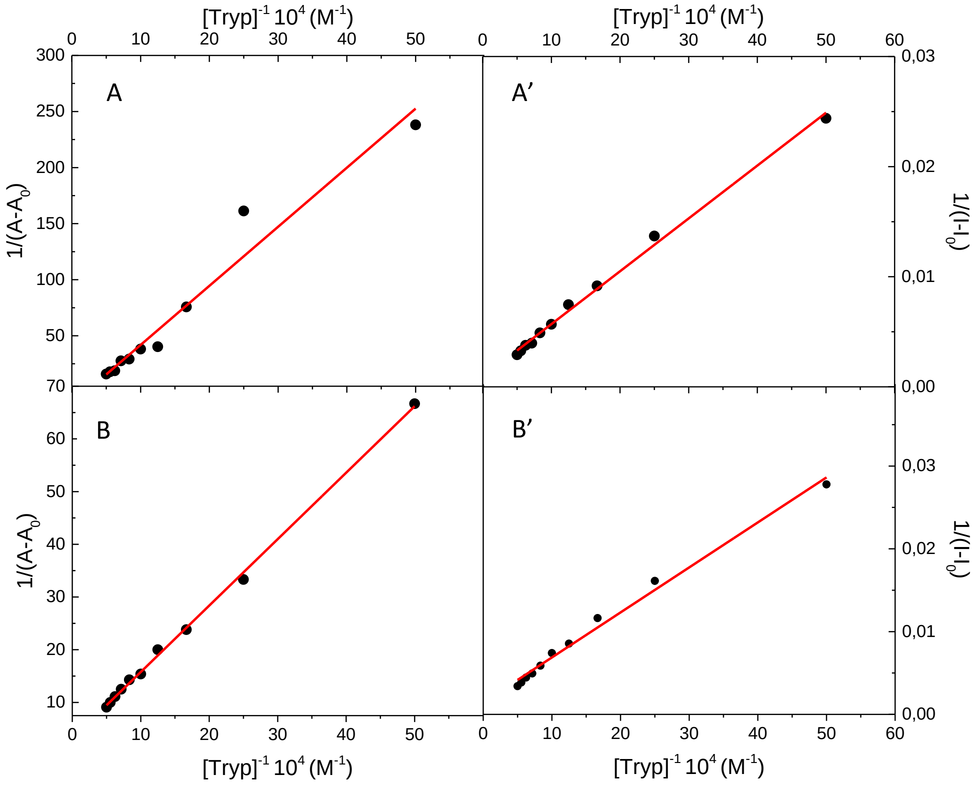

Figure S2. The Benesi-Hildebrand dependence (1/(A-A$_0$) *vs.* [Tryp]$^{-1}$) and (1/(I-I$_0$) *vs.* [Tryp]$^{-1}$) for **I** (**A**, **A′**) and **II** (**B**, **B′**) in phosphate buffer solutions containing different concentration of Tryp.

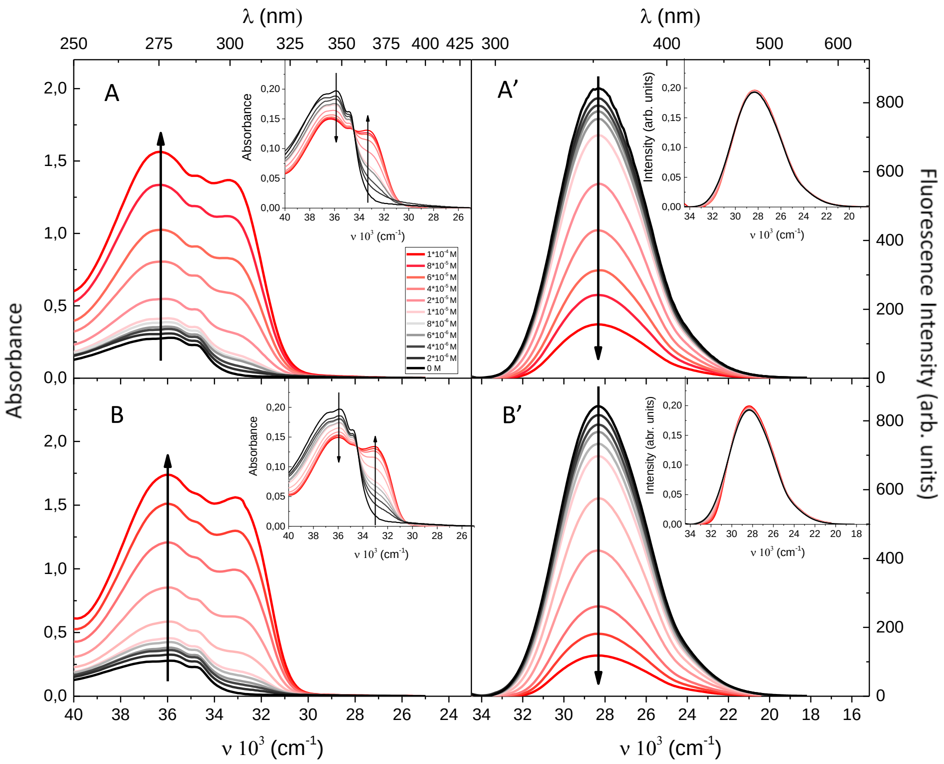

Figure S3 Absorption and fluorescence spectra ($\lambda_{exc.}$=280 nm) of Tryp (c=$5{\cdot}10^{-5}$ M) in phosphate buffer solutions containing different concentration of **I** (**A**, **A'**) and **II** (**B**, **B'**) (0 M, $2.0{\cdot}10^{-6}$ M, $4.0{\cdot}10^{-6}$ M, $6.0{\cdot}10^{-6}$ M, $8.0{\cdot}10^{-6}$ M, $1.0{\cdot}10^{-5}$ M, $1.2{\cdot}10^{-5}$ M, $1.4{\cdot}10^{-5}$ M, $1.6{\cdot}10^{-5}$ M, $1.8{\cdot}10^{-5}$ M, $2.0{\cdot}10^{-5}$ M). The normalized LW absorption and fluorescence band (by scaling the area under the spectrum to be equal to unity) are presented in inserts.

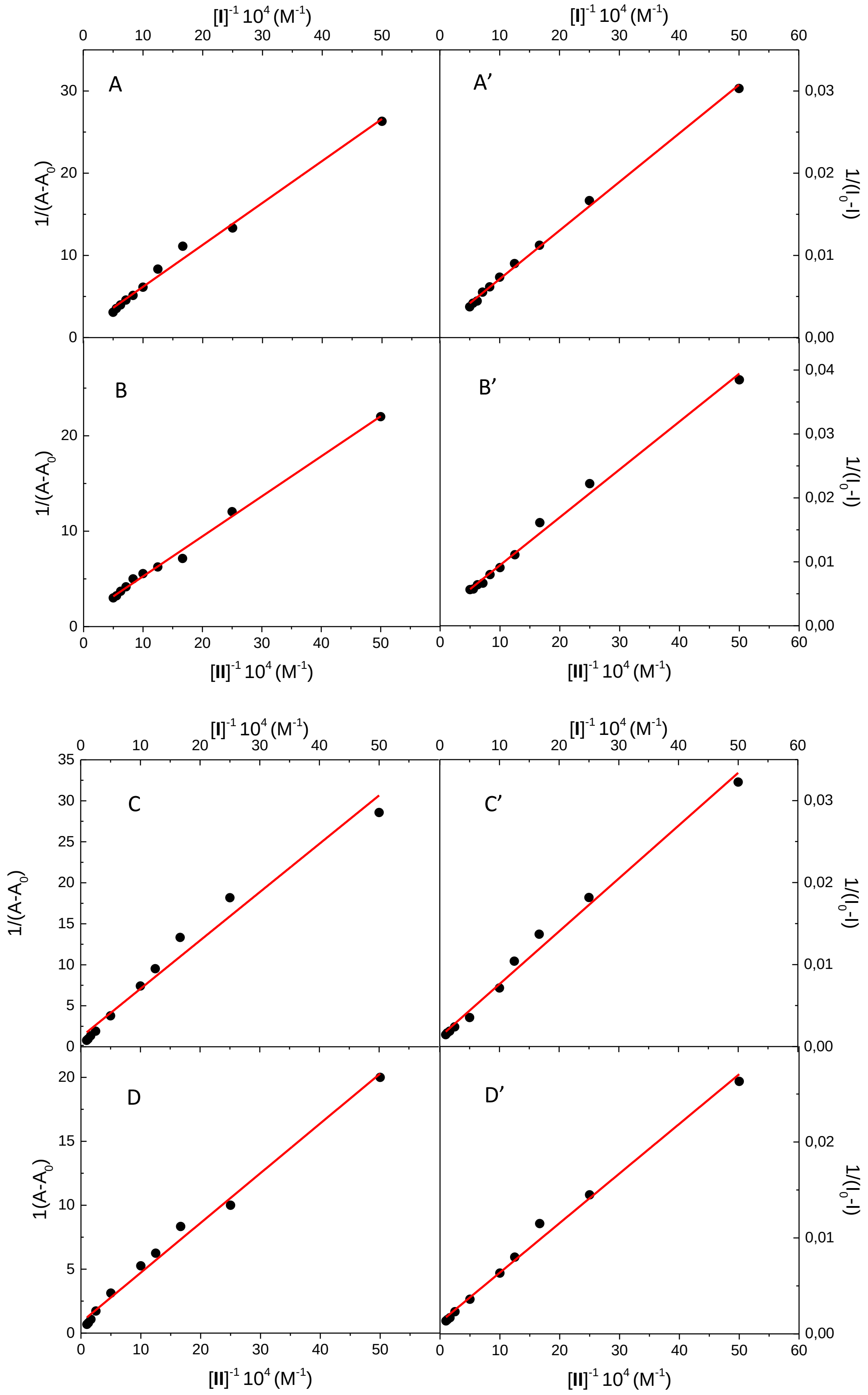

Figure S4 The Benesi-Hildebrand dependence $1/(A\text{-}A_0)$ *vs.* $[\text{Molecule}]^{-1}$ (**A**, **B**, **C**, **D**) and $1/(I_0\text{-}I)$ *vs.* $[\text{Molecule}]^{-1}$ (**A′**, **B′**, **C′**, **D′**) for BSA-**I** (**A**, **A′**), BSA-**II** (**B**, **B′**), Tryp-**I** (**C**, **C′**) and Tryp-**II** (**D**, **D′**) systems in phosphate buffer solutions containing different concentration of investigated molecules.

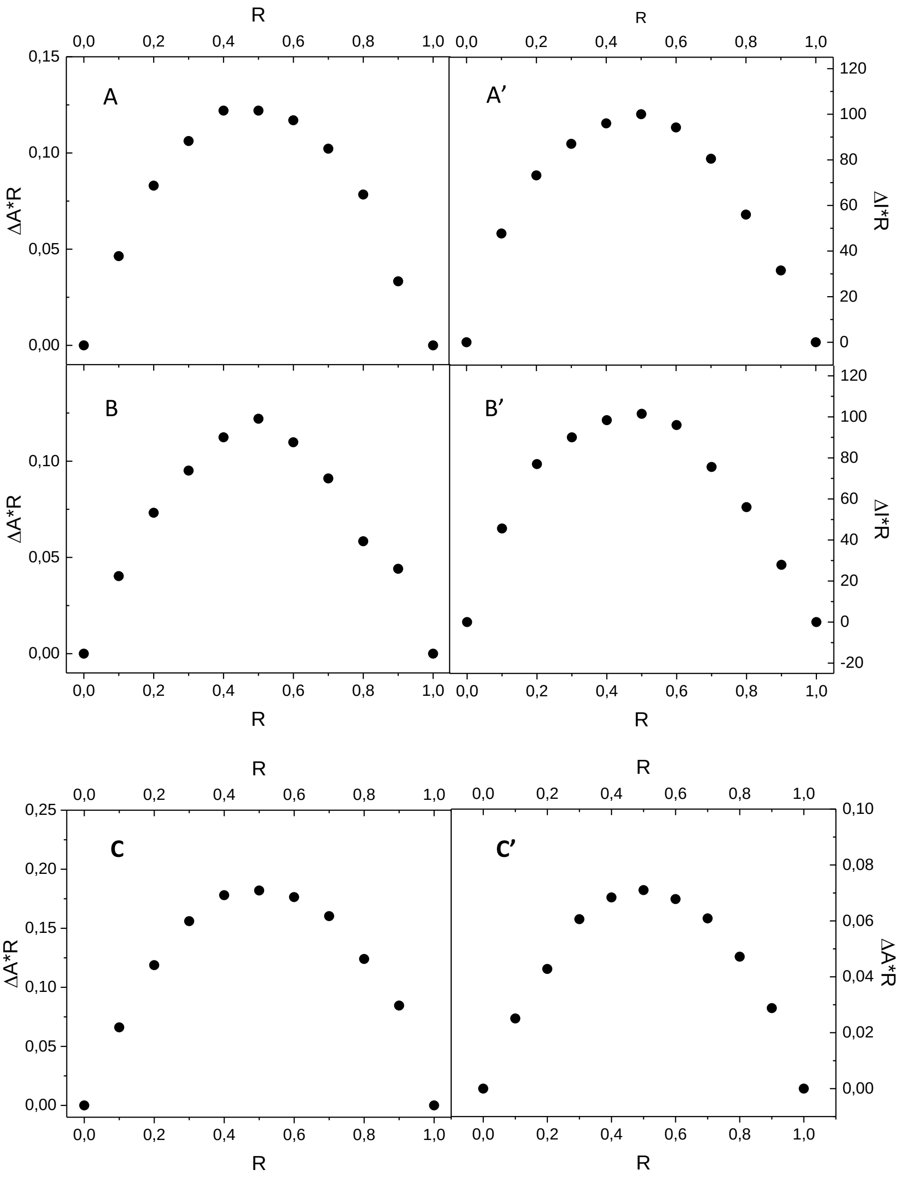

Figure S5 Job plots of the investigated BSA-**I** (**A**, **A′**), BSA-**II** (**B**, **B′**), Tryp-**I** (**C**) and Tryp-**II** (**C′**) systems prepared by using steady-state absorption (**A**, **B**, **C**) and fluorescence (**A′**, **B′**, **C′**) data.
